# Tracing of Afferent Connections in the Zebrafish Cerebellum Using Recombinant Rabies Virus

**DOI:** 10.3389/fncir.2019.00030

**Published:** 2019-04-24

**Authors:** Ryuji Dohaku, Masahiro Yamaguchi, Naoyuki Yamamoto, Takashi Shimizu, Fumitaka Osakada, Masahiko Hibi

**Affiliations:** ^1^Division of Biological Science, Graduate School of Science, Nagoya University, Nagoya, Japan; ^2^Laboratory of Cellular Pharmacology, Graduate School of Pharmaceutical Sciences, Nagoya University, Nagoya, Japan; ^3^Department of Animal Sciences, Graduate School of Bioagricultural Sciences, Nagoya University, Nagoya, Japan; ^4^Laboratory of Organogenesis and Organ Function, Bioscience and Biotechnology, Nagoya University, Nagoya, Japan

**Keywords:** cerebellum, afferents, connections, purkinje cells, granule cells, rabies virus, zebrafish

## Abstract

The cerebellum is involved in some forms of motor coordination and learning, and in cognitive and emotional functions. To elucidate the functions of the cerebellum, it is important to unravel the detailed connections of the cerebellar neurons. Although the cerebellar neural circuit structure is generally conserved among vertebrates, it is not clear whether the cerebellum receives and processes the same or similar information in different vertebrate species. Here, we performed monosynaptic retrograde tracing with recombinant rabies viruses (RV) to identify the afferent connections of the zebrafish cerebellar neurons. We used a G-deleted RV that expressed GFP. The virus was also pseudotyped with EnvA, an envelope protein of avian sarcoma and leucosis virus (ALSV-A). For the specific infection of cerebellar neurons, we expressed the RV glycoprotein (G) gene and the envelope protein TVA, which is the receptor for EnvA, in Purkinje cells (PCs) or granule cells (GCs), using the promoter for *aldolase Ca* (*aldoca*) or *cerebellin 12* (*cbln12*), respectively. When the virus infected PCs in the *aldoca* line, GFP was detected in the PCs’ presynaptic neurons, including GCs and neurons in the inferior olivary nuclei (IOs), which send climbing fibers (CFs). These observations validated the RV tracing method in zebrafish. When the virus infected GCs in the *cbln12* line, GFP was again detected in their presynaptic neurons, including neurons in the pretectal nuclei, the nucleus lateralis valvulae (NLV), the central gray (CG), the medial octavolateralis nucleus (MON), and the descending octaval nucleus (DON). GFP was not observed in these neurons when the virus infected PCs in the *aldoca* line. These precerebellar neurons generally agree with those reported for other teleost species and are at least partly conserved with those in mammals. Our results demonstrate that the RV system can be used for connectome analyses in zebrafish, and provide fundamental information about the cerebellar neural circuits, which will be valuable for elucidating the functions of cerebellar neural circuits in zebrafish.

## Introduction

The cerebellum is involved not only in smooth and skillful movements but also in cognitive and emotional functions, such as fear conditioning and reward expectations (Ito et al., 1982; Raymond et al., 1996; Yoshida et al., 2004; Ito, 2006; Glickstein, 2007; Voogd, 2014; Strata, 2015; Adamaszek et al., 2017; Matsuda et al., 2017; Wagner et al., 2017; Wylie et al., 2018; Schmahmann, 2019). However, little is known about the mechanisms by which the cerebellum elicits these functions. Comparative studies of the cerebellum of different vertebrate species, particularly those having a simple cerebellar structure, may provide clues for understanding the general functions of the cerebellum. The organization of the cerebellum is generally conserved between mammals and teleosts, including zebrafish (Nieuwenhuys, 1967; Finger, 1983; Meek, 1992; Butler and Hodos, 1996; Altman and Bayer, 1997; Köster and Fraser, 2001, 2006; Volkmann et al., 2008, 2010; Bae et al., 2009; Kani et al., 2010; Tanabe et al., 2010; Wullimann et al., 2011; Hibi and Shimizu, 2012; Takeuchi et al., 2015, 2017; Hibi et al., 2017). The zebrafish brain is a particularly versatile model for studying the functions and development of the vertebrate brain, including the cerebellar neural circuits, since it is smaller than the mammalian brain, and the zebrafish body is transparent during the early larval stages (and at late larval stages in a pigment-less mutant background). In addition, Ca^2+^ imaging and optogenetic manipulation can be used to study zebrafish neural circuits (Matsui et al., 2014a; Kawashima et al., 2016; Song et al., 2016; Cong et al., 2017; Knogler et al., 2017; Matsuda et al., 2017).

**Figure 1:**
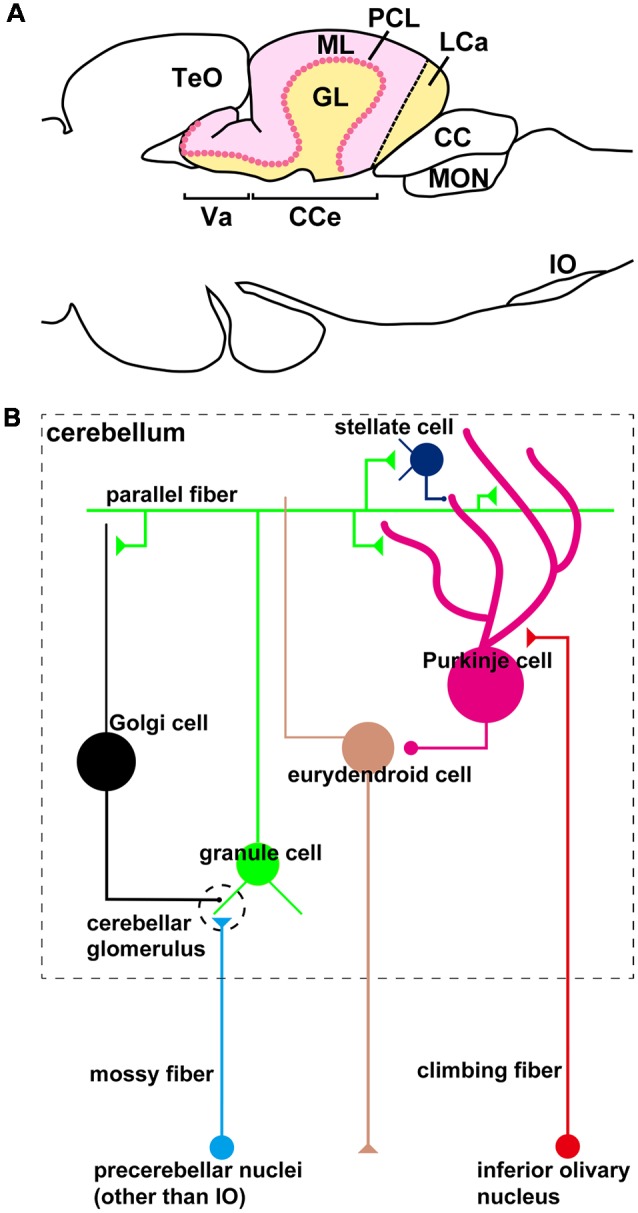
The zebrafish cerebellum and cerebellar neural circuits. **(A)** Schematic illustration of the zebrafish cerebellum. Sagittal section image. **(B)** Schematic illustration of the zebrafish cerebellar neural circuits. The neural circuits within the cerebellum are marked by a dashed box.

**Figure 2:**
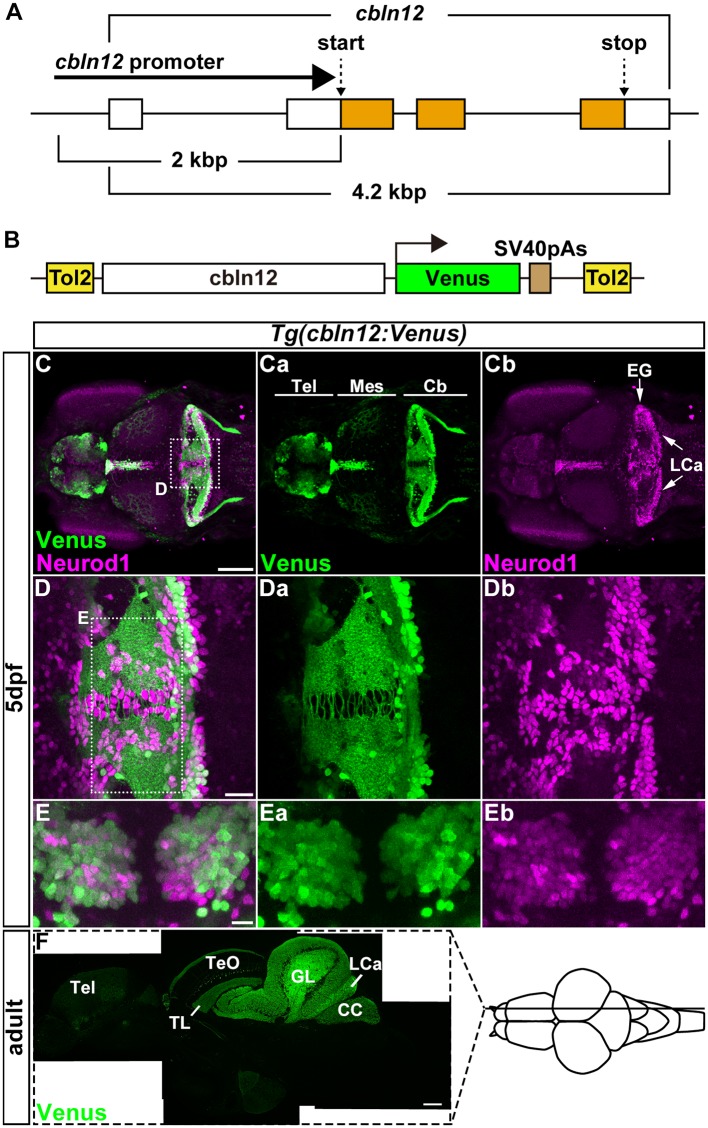
The *cbln12* promoter drives transgene expression in granule cells (GCs). **(A)** Genomic structure of the *cbln12* gene. *cbln12* has four exons and a total length of about 4.2 kbp. The start codon is in exon 2, and the stop codon is in exon 4. We isolated a 2-kbp fragment extending upstream from the start codon (*cbln12* promoter). **(B)** Schematic drawing of the Tol2 plasmid used to express Venus in the GCs. We fused the *cbln12* promoter to Venus cDNA and inserted it into a Tol2 vector. This construct was used to establish *Tg(cbln12:Venus)* lines. **(C–E)** Immunostaining of 5-days post fertilization (dpf) *Tg(cbln12:Venus)* larvae with anti-GFP (green) and anti-Neurod1 antibodies (magenta). Venus **(Ca,Da,Ea)**, Neurod1 **(Cb,Db,Eb)**, and merged images **(C,D,E)** are shown. Dorsal views with rostral to the left **(C,Ca,Cb,D,Da,Db)** or to the top **(E,Ea,Eb)**. **(D,E)** High-magnification images of the boxes in **(C,D)**. Venus was expressed in the Tel, the TL, and the Cb. In the medial region and the caudal edge of the Cb, some Neurod1^+^ cells did not express Venus **(D)**. These were probably immature GCs. In the ventral region of the Cb **(E)**, most of the Neurod1^+^ neurons co-expressed Venus **(E)**. **(F)** Sagittal section of an adult *Tg(cbln12:Venus)* brain was stained with an anti-GFP antibody. Scale bars: **(C)** 100 μm, **(D)** 20 μm, **(E)** 10 μm, and **(F)** 200 μm.

The organization of the zebrafish cerebellum has been characterized in detail (Wullimann et al., 1996; Bae et al., 2009). It has three domains: the rostral-most region is called the valvula cerebelli (Va), the middle region is called the corpus cerebelli (CCe); and the lateral and caudal regions are functionally linked and called the eminentia granularis (EG) and lobus caudalis cerebelli (LCa), respectively (Figures 1A, 2C). As in the mammalian cerebellum, the Va and CCe are composed of three layers, called the molecular layer (ML), the Purkinje cell layer (PCL), and the granular layer (GL), from superficial to deep. On the other hand, the EG and the LCa have a distinct layer structure in which the GL is located at their surface (Figure 1A). There are several types of neurons in the zebrafish cerebellum, including granule cells (GCs), Purkinje cells (PCs), Golgi cells (GoCs), and eurydendroid cells (ECs); the latter are teleost-specific projection neurons with connections similar to those of neurons in the deep cerebellar nuclei in mammals (Figure 1B). There are two types of afferent fibers to the cerebellum: climbing fibers (CFs) and mossy fibers (MFs). The CFs and MFs form synapses with PCs and GCs, respectively (Figure 1B). The CFs and MFs are projections of precerebellar neurons, and the nucleus containing these neurons is called the precerebellar nucleus. The information from the MFs is conveyed by the axons of GCs (called parallel fibers, PFs) to the dendrites of the PCs. The information from the CFs and MFs is integrated in the PCs. The ECs receive inputs from the PC axons and probably also from the PFs, and send outputs outside the cerebellum. Although the local circuit structure in the zebrafish cerebellum has been well investigated, its afferent and efferent connections with other brain areas have not been fully elucidated.

In mammals, the CFs originate from neurons in the contralateral side of the inferior olivary nucleus (IO; Altman and Bayer, 1997; Sugihara, 2006), and the MFs originate from neurons in various brain regions, such as the pontine nuclei, vestibular nuclei, external (accessory) cuneate nuclei, lateral reticular nuclei, and spinal cord (Hibi and Shimizu, 2012). The CFs in teleosts, including zebrafish, also originate largely from the contralateral IO neurons (Finger, 1978; Meek et al., 1986a,b; Wullimann and Northcutt, 1988, 1989; Xue et al., 2004; Folgueira et al., 2006; Takeuchi et al., 2015), suggesting that the CFs are conserved between mammals and teleosts. In contrast, there are variations in the precerebellar neurons for the MFs between mammals and teleosts. Neurons in several pretectal regions, such as the central pretectal nucleus (CPN), the intercalated pretectal nucleus (Pi), and the paracommissural nucleus (PCN), are reported to extend their axon to the cerebellum as a MF in zebrafish (Yáñez et al., 2018). The connections of the cerebellum have also been studied in other teleost species (Finger, 1978; Wullimann and Northcutt, 1989; Ikenaga et al., 2002, 2005; Xue et al., 2004; Folgueira et al., 2006; Huesa et al., 2006). For instance, injecting the lipophilic dye DiI or horseradish peroxidase (HRP) into the cerebellum of rainbow trout and catfish revealed that the cerebellum receives afferent fibers from neurons in the ventral accessory optic nucleus (VAO), the CPN, the PCN, the intermediate pretectal nucleus (IN), the torus semicircularis (TS), the nucleus lateralis valvulae (NLV), the central gray (CG), the locus coeruleus (LC), the octavolateral region, and the IO (Finger, 1978; Folgueira et al., 2006). Although DiI and HRP are useful tools for connectome analyses, they do not target specific types of cerebellar neurons or provide evidence for synaptic connections with particular cerebellar neurons. To determine the precise locations of the precerebellar neurons (nuclei) for the MFs in teleosts, cell-type-specific and synapse-dependent tracing methods are needed.

Retrograde tracing methods using recombinant rabies virus (RV) have been used to label presynaptic (afferent) neurons across the whole brain (Wickersham et al., 2007a,b; Osakada and Callaway, 2013). Wild-type RV has five genes in its genome. Among them, the gene for the envelope glycoprotein (G) is needed for the retrograde transport and infection of the viral particles (Mebatsion et al., 1996; Etessami et al., 2000). In the monosynaptic RV tracing method, the G gene in the RV genome is replaced by the GFP gene (RVΔG-GFP). Furthermore, the virus particles are pseudotyped with the envelope protein of the avian sarcoma leucosis virus (ALSV) called EnvA (EnvA-RVΔG-GFP). This engineered virus can only infect cells that express the EnvA receptor TVA or its fusion protein. When the infected cells express the RV protein G, functional viral particles are generated, transported, and infect presynaptic neurons in a synapse-dependent manner. If the infected cells do not express G, the virus cannot be transferred to their presynaptic neurons. Thus, only monosynaptic transmission from the starter neurons expressing both TVA and G takes place, and the directly afferent neurons are labeled by GFP expression. This method has been applied extensively for connectome analyses in mammals (Schwarz et al., 2015; Stephenson-Jones et al., 2016; Beier et al., 2017), but not for zebrafish. In this study, we optimized the RV tracing method for zebrafish and analyzed the cerebellar afferent connections in this animal.

## Materials and Methods

### Zebrafish

Wild-type zebrafish (*Danio rerio*) with the Oregon AB genetic background (RRID:ZIRC_ZL1) were used. The zebrafish were maintained in environmentally controlled rooms at the Bioscience and Biotechnology Center, Nagoya University, on a 14–10 h light-dark cycle (light 9 am to 11 pm; dark 11 pm to 9 am) at 28.5°C.

### Generation of Transgenic Zebrafish Lines

To establish transgenic (Tg) lines that express transgenes in GCs, we used a regulatory element for the *cerebellin12* (*cbln12*) gene (NM_001243318 in GenBank, *cbln12* promoter; Takeuchi et al., 2017). An approximately 2-kbp genomic fragment upstream of the translation initiation site of *cbln12* was amplified from zebrafish genomic DNA by PCR with the following primers:5′-GGGGACAAGTTTGTACAAAAAAGCAGGCTCGATTCTGTGTGCTTTGTTT-3′, 5′-GGGGACAACTTTTGTATACAAAGTTGTACAACTTCCAAAATCTCTGA-3′ (the underlined sequences are *attB1*/*attB5r* sites) and subcloned using the BP reaction of the Gateway system (Thermo Fisher) to generate an entry vector pENTR-L1-cbln12p-R5. pENTR-L1-cbln12p-R5 and another entry vector containing the Venus cDNA and the polyadenylation site (pAS) of SV40 from pCS2+Venus (Nagai et al., 2002; pENTR-L5-Venus-pAS-L2) were subcloned into the Tol2 vector pT2KDest-RfaF (Nojima et al., 2010) by the LR reaction of the Gateway system (pT2K-cbln12-Venus-pAS). To express TVA-mCherry and G in PCs or GCs, we subcloned the 5-kbp *aldolase Ca* (*aldoca*) promoter or the *cbln12* promoter fragment, and the TVA-mCherry cDNA (encoding a fusion protein of TVA950 and mCherry) from pAAV-Ef1a-DIO-TVA-mCherry (Watabe-Uchida et al., 2012) or the G (B19G) cDNA from pcDNA-B19G (Osakada et al., 2011) into pCS2+. The *aldoca:TVA-mCherry-pAS*, *aldoca:G-pAS*, *cbln12:TVA-mCherry-pAS*, and *cbln12:G-pAS* cassettes were amplified by PCR with primers containing *attB1*/*att*B2 sites, and subcloned to generate entry vectors using the Gateway system. These entry vectors were used for Gateway-mediated subcloning into the Tol2 vectors pT2ALR-Dest and pBleeding Heart (pBH)-R1-R2, which were derived from pT2AL200R150G (Urasaki et al., 2006) and pBH-R4-R2 (van Ham et al., 2010), respectively. The *aldoca:TVA-mCherry-pAS* and *cbln12:TVA-mCherry-pAS* cassettes were subcloned into pT2ALR-Dest, while the *aldoca:-G-pAS* and *cbln12:G-pAS* cassettes were subcloned into pBH-R1-R2, which contained the regulatory element *myosin, light chain 7, regulatory* (*myl7*), the mCherry cDNA, and the pAS. To make Tg fish, 25 pg of the Tol2 plasmids and 25 pg of transposase capped RNA were injected into one-cell-stage embryos. The F1 generation of Tg fish was identified by the expression of TVA-mCherry in the PCs or GCs at 5 days post fertilization (dpf), or by the expression of mCherry in the heart at 2 dpf. Tg fish expressing both TVA-mCherry and G in the PCs or GCs and mCherry in the heart were obtained by crossing Tg fish expressing TVA-mCherry and Tg fish expressing G in the cerebellum and mCherry in the heart.

### Production of G-Deleted Rabies Virus (RV) Solution

A G-deleted rabies viral vector encoding GFP (SAD-B19ΔG-GFP) was generated as described previously (Osakada and Callaway, 2013). In brief, RVΔG-GFP was recovered by transfecting B7GG cells with the RV genomic plasmids pSAD-B19ΔG-GFP, pcDNA-SAD-B19N, pcDNA-SAD-B19P, pcDNASAD-B19L, and pcDNA-SAD-B19G, and cultured in a humidified atmosphere of 3% CO_2_ at 35°C. For the pseudotyping of SAD-B19ΔG-GFP with EnvA, BHK-EnvA cells were infected with unpseudotyped SAD-B19ΔG-GFP RV, washed with phosphate buffered saline (PBS), treated with 0.25% trypsin-EDTA, and replated on new dishes. For injection, the EnvA-pseudotyped SAD-B19ΔG viruses were produced in 10 150-mm dishes in a humidified atmosphere of 3% CO_2_ at 35°C, passed through a 0.45-μm filter, and concentrated by two rounds of ultracentrifugation. The EnvA-pseudotyped RV were titrated with HEK293-TVA cells. The titer of EnvA-pseudotyped SAD-B19ΔG-GFP was 1.8 × 10^8^ infectious units/mL. Because contamination by unpseudotyped RV in the viral solution would cause TVA-independent non-specific infection, we confirmed that there was no sign of contamination by injecting the pseudotyped RV into wild-type mouse brains lacking TVA expression. We also did not observe GFP expression when we injected the RV solution into wild-type zebrafish.

### Rabies Virus (RV) Injection

The adult Tg zebrafish (older than 3 months post-fertilization) were anesthetized in 0.02% tricaine methanesulfonate (MS-222) and embedded on sponge. A hole was made in the skull bone over the cerebellum by a drill (MINITOR, Cat# RPM-25S). About 50–100 nL of the RV (EnvA-RVΔG-GFP) solution was injected through the hole using a pneumatic microinjector (PV820, WPI). The injected fish were kept at 34–35.5°C for 5–10 days.

### Immunohistochemistry

**Figure 3:**
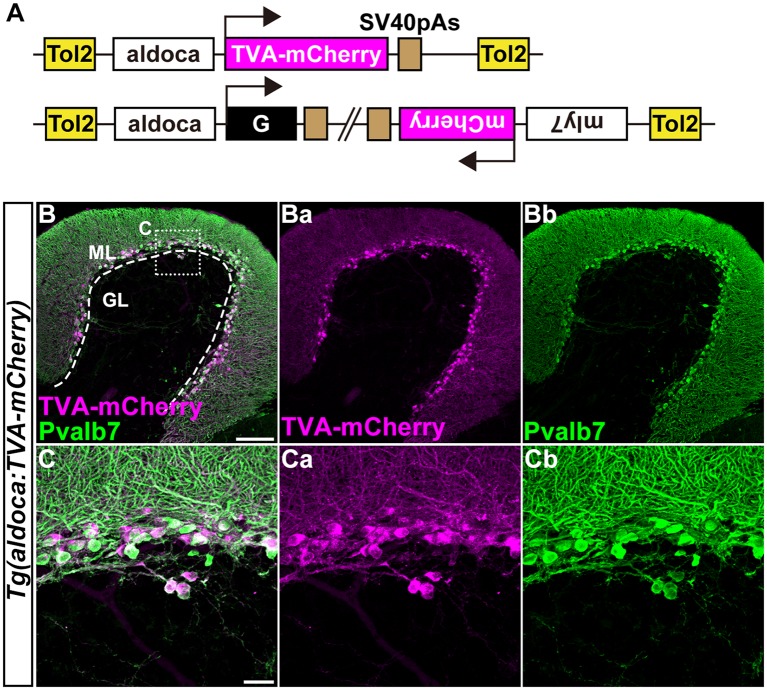
Transgenic lines for tracing Purkinje cell (PC) afferents. **(A)** Schematic drawing of the Tol2 plasmids used to express TVA-mCherry or rabies virus glycoprotein (G). The approximately 5-kbp *aldoca* promoter was used to express transgenes specifically in PCs. To identify Tg fish harboring the G transcription unit, the Tol2 vector pBH (Bleeding Heart vector), which expresses mCherry in the heart was used. **(B,C)** Immunostaining of sagittal sections of the adult *Tg(aldoca:TVA-mCherry)* fish cerebellum with anti-mCherry (anti-DsRed, magenta) and anti-parvalbumin7 (anti-Pvalb7, green) antibodies. **(C)** High-magnification images of the box in **(B)**. TVA-mCherry **(Ba,Ca)**, Pvalb7 **(Bb,Cb)**, and merged images **(B,C)** are shown. PCs that expressed TVA-mCherry also expressed Pvalb7. Scale bars: **(B)** 100 μm, and **(C)** 20 μm.

**Figure 4:**
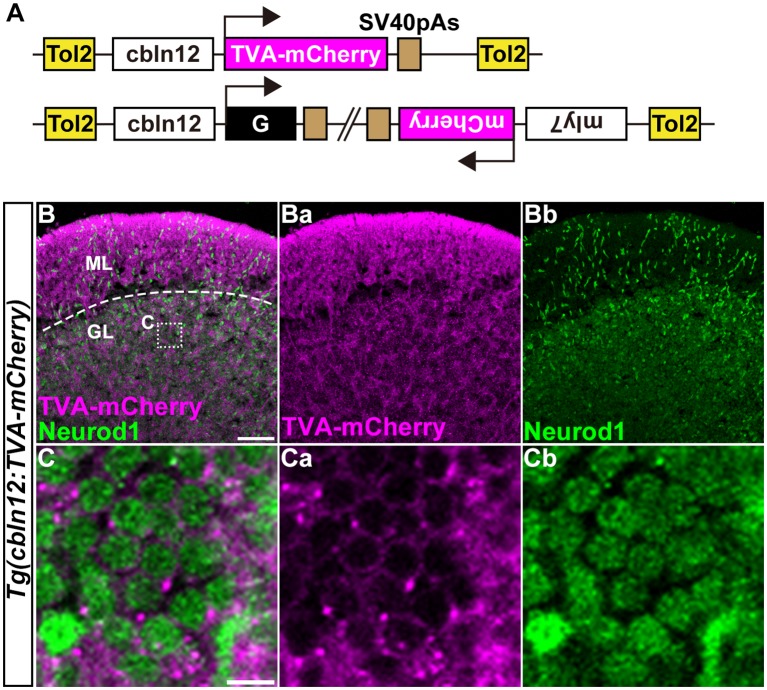
Transgenic lines for tracing GC afferents. **(A)** Schematic drawing of the Tol2 plasmids used to express TVA-mCherry or RVG. The *cbln12* promoter was used to express transgenes in GCs. pBH was used to identify G-expressing fish. **(B,C)** Immunostaining of sagittal sections of the adult *Tg(cbln12:TVA-mCherry)* cerebellum with anti-mCherry (magenta) and anti-Neurod1 (green) antibodies. **(C)** High-magnification images of the box in **(B)**. TVA-mCherry **(Ba,Ca)**, Neurod1 **(Bb,Cb)**, and merged images **(B,C)** are shown. Note that TVA-mCherry was located on the cell membrane of Neurod1^+^ GCs **(C)**. Scale bars: **(B)** 50 μm, and **(C)** 5 μm.

**Figure 5:**
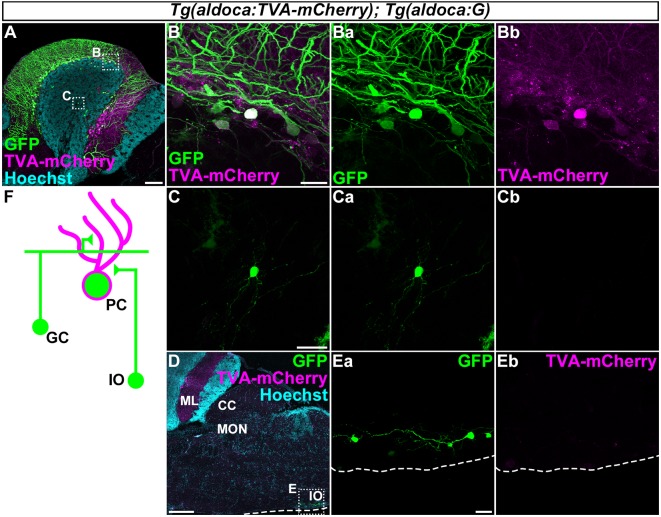
Tracing of the presynaptic neurons of PCs using the RV method. A solution of pseudotyped RV was injected into the left side of the cerebellum of adult *Tg(aldoca:TVA-mCherry); Tg(aldoca:G)* fish. The injected fish were reared at 34–35.5°C for 5 days. The brains were then harvested, fixed, and subjected to immunostaining. **(A–E)** Staining of sagittal sections with anti-mCherry (magenta) and anti-GFP (green) antibodies, and the nuclear marker Hoechst (cyan). **(B,C,Ea,Eb)** Higher-magnification images of the boxes in **(A,D)**. GFP **(Ba,Ca,Ea)**, TVA-mCherry **(Bb,Cb,Eb)**, and merged images **(B,C)** are shown. GFP was observed in the molecular layer (ML) and PC layer (PCL; **A**). GFP was detected in the TVA-mCherry^+^ PCs **(B)**. GFP but not TVA-mCherry was also detected in neurons in the granular layer (GL; **C**) and the inferior olivary (IOs; **E**). **(F)** Schematic summary of the PC afferent tracing results. Scale bar: **(A)** 100 μm, **(B)** 20 μm, **(C)** 20 μm, **(D)** 200 μm, and **(E)** 20 μm.

**Figure 6:**
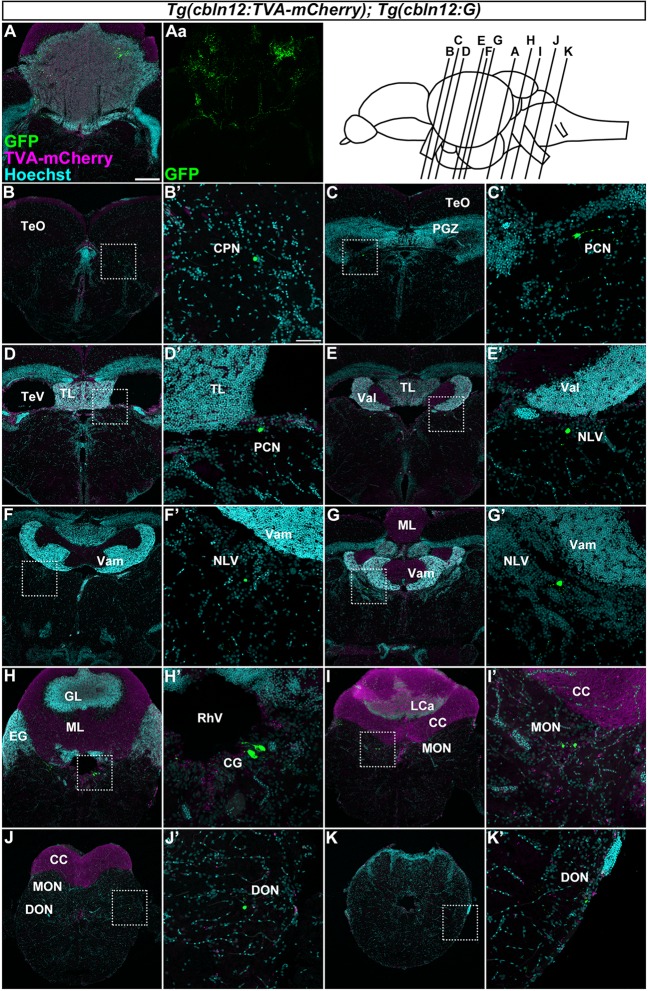
Tracing of mossy fibers (MFs) using the RV method. The RV solution was injected into the left side of the cerebellum of adult *Tg(cbln12:TVA-mCherry); Tg(cbln12:G)* fish. The injected fish were then reared at 34–35.5°C for 10 days. The brains were harvested, fixed, and subjected to immunostaining. **(A–K)** Staining of brain cross sections with anti-mCherry (magenta) and anti-GFP (green) antibodies, and Hoechst (cyan). In the injected regions, GFP and TVA-mCherry were detected in neurons and neurites in the cerebellum **(A,Aa)**. GFP but not TVA-mCherry was detected in neurons in the CPN **(B)**, the paracommissural nucleus (PCN; **C,D**), the nucleus lateralis valvulae (NLV; **E–G)**, the GC **(H)**, the medial octavolateralis nucleus (MON; **I**), and the descending octaval nucleus (DON; **J,K**). Scale bar: **(A)** 200 μm, **(B’)** 50 μm. The magnification in **(B–K)** and **(C’–K’)** was the same as in **(A)** and **(B’)**, respectively.

Larvae at 5-dpf or the adult brain were fixed overnight at 4°C in 4% paraformaldehyde (PFA) in PBS (pH 7.4). Adult brains were sectioned at 14 μm (Figures 2–4) or 25 μm (Figures 5, 6) by a cryostat (CM1860, Leica). Larvae and cryostat sections were immunostained as described previously (Bae et al., 2009; Kani et al., 2010). For immunostaining, anti-GFP (1:1,000, rat, Nacalai Tesque, Cat# 04404-84 RRID:AB_10013361) for Venus or GFP, anti-DsRed (1:1,000, rabbit, Clontech Laboratories, Inc., Cat# 632496 RRID:AB_10014383) for mCherry, anti-Neurod1 (1:500, mouse, ascites), and anti-parvalbmin7 (1:1,000, mouse monoclonal, ascites; Bae et al., 2009; Kani et al., 2010) antibodies were used. Alexa Fluor 488 goat anti-rat IgG (H + L, Thermo Fisher Scientific Cat# A-11006, RRID:AB_2534074), CF488A goat anti-rat IgG (H + L, Biotium Cat# 20023, RRID:AB_10557403), CF488A goat anti-mouse IgG (H + L, Biotium Cat# 20018-1, RRID:AB_10853449), Alexa Fluor 568 goat anti-rabbit IgG (H + L, Molecular Probes Cat# A-11011, RRID:AB_143157), CF568 goat anti-rabbit IgG (H + L, Biotium Cat# 20103, RRID:AB_10558012), and CF568 goat anti-mouse IgG (H + L, Biotium Cat# 20301-1) were used as the secondary antibody. Sections were also stained with a cell nucleus marker Hoechst 33342. Fluorescent images were captured with an LSM700 confocal laser-scanning microscope (Zeiss). Images were constructed from Z-stack sections by the 3D projection program associated with the microscope (Zen, Zeiss) and ImageJ. The figures were constructed using Adobe Photoshop and Adobe illustrator. Adjustments of the brightness and contrast of digital images were applied equally to all of the images within a figure.

## Results

### Identification of a GC-Specific Promoter/Enhancer

For retrograde tracing with the pseudotyped RV, TVA or its fusion protein had to be expressed specifically in cerebellar neurons. Although PC-specific promoter/enhancer elements have been reported (Tanabe et al., 2010; Matsui et al., 2014a), GC-specific promoter/enhancer elements have not. We previously revealed that *cerebellin 12* (*cbln12*), a zebrafish ortholog of *Cerebellin1* in mammals, is specifically expressed in GCs in the cerebellum (Takeuchi et al., 2017). We isolated a genomic fragment of about 2-kbp extending upstream from the translational initiation site of the *cbln12* gene (referred to as the *cbln12* promoter), containing the transcription start site and the first exon and intron, and used it to express the fluorescent protein Venus in GCs (Figures 2A,B). We established zebrafish transgenic (Tg) lines using the Tol2 transposon-mediated transgenic method (Kawakami et al., 2004). All of the *Tg(cbln12:Venus)* lines expressed Venus in the telencephalon, the mesencephalic tectum, and the cerebellum (Figure 2C). In the Tg larvae, Venus was detected in both the soma and neurites of the neurons in the cerebellum. All of the Venus^+^ somata in the cerebellum were also positive for neural differentiation 1 (Neurod1), a marker of immature and mature GCs in the cerebellum (Mueller and Wullimann, 2003; Kani et al., 2010; Matsuda et al., 2017), suggesting that the Venus^+^ cells were GCs. In addition, there were Neurod1-positive Venus-negative cells near the midline of the CCe and the caudal edge of the LCa (Figure 2D). These cells were probably differentiating immature GCs that were derived from the *atoh1*-expressing GC progenitors located in the midline and the caudal edge of the cerebellum (Kani et al., 2010). The Venus and Neurod1 expression almost completely overlapped in the medial regions of the CCe, where mature GCs are present (Bae et al., 2009; Kani et al., 2010; Figure 2E), indicating that Venus was expressed in mature GCs. This finding was consistent with a previous report that *cbln12* is expressed in differentiated GCs (Takeuchi et al., 2017). Sagittal sections of the adult Tg brain showed that Venus was detected in cell bodies in the torus longitudinalis (TL) in the tectum, in the GL and LCa in the cerebellum where the GC somata were located, and in nerve fibers in the stratum marginale (SM) of the tectum, in the ML of the CCe, and in the crista cerebellaris (CC) in the dorsal hindbrain where the GC axons were present (Figure 2F). All of these observations indicated that the *cbln12* promoter could drive transgene expression in the mature GCs in zebrafish.

### Establishment of Tg Lines for RV Tracing

We previously showed that the approximately 5-kbp genomic region upstream from the translational initiation site of the (*aldoca*) gene (the *aldoca* promoter) can drive the expression of transgenes specifically in PCs (Tanabe et al., 2010). By using the *aldoca* promoter or the *cbln12* promoter, we established Tg lines that expressed G and TVA-mCherry, which is a fusion protein of TVA950 and mCherry, in PCs or GCs (Figures 3, 4). For each cell type, we constructed two independent Tol2 plasmids to express TVA-mCherry or G from separate transgenes (Figures 3A, 4A). Although the TVA-mCherry expression is detected by its fluorescence, G cannot be detected without additional markers. To identify Tg lines harboring the G transgene, we inserted the G transcription unit into a Tol2 plasmid (pBleeding Heart: pBH) that contained the promoter for *myosin, light chain 7, regulatory* (*myl7*) and mCherry cDNA, that drives mCherry expression in the heart (van Ham et al., 2010). By Tol2-mediated transgenesis (Kawakami et al., 2004), we established *Tg(aldoca:TVA-mCherry)* and *Tg(aldoca:G, myl7:mCherry)* to trace the afferents to PCs, and *Tg(cbln12:TVA-mCherry)* and *Tg(cbln12:G, myl7:mCherry)* to trace the afferents to GCs. In the cerebellum of the adult *Tg(aldoca:TVA-mCherry)* fish, the TVA-mCherry expression completely overlapped with that of parvalbumin7 (Pvalb7), a marker of PCs in zebrafish (Bae et al., 2009; *n* = 4, Figures 3B,C). In the cerebellum of adult *Tg(cbln12:TVA-mCherry)* fish, TVA-mCherry was detected in the GL and the ML, where the axons and somata of GCs are located (Figure 4B). TVA-mCherry was detected on the cell-body surface of the Neurod1^+^ GCs in the GL of the *Tg(cbln12:TVA-mCherry)* cerebellum (*n* = 4, Figure 4C). Expression of TVA-mCherry was similar to that of Venus in the *Tg(cbln12:Venus)* fish (Supplementary Figure S1). These observations indicated that *Tg(aldoca:TVA-mCherry)* and *Tg(cbln12:TVA-mCherry)* fish expressed TVA-mCherry specifically in PCs and GCs in the cerebellum, respectively, which would confer the infectivity of the pseudotyped RV to these neurons. For retrograde tracing, we crossed the *Tg(aldoca:TVA-mCherry)* and *Tg(cbln12:TVA-mCherry)* fish with *Tg(aldoca:G, myl7:mCherry)* and *Tg(cbln12:G, myl7:mCherry)*, respectively. The larvae expressing mCherry in both cerebellar neurons (PCs or GCs) and the heart were selected and reared to adulthood for RV infection.

### Development of an RV Tracing Method in Zebrafish

First, to validate our strategy, we injected the recombinant pseudotyped RV (EnvA-RVΔG-GFP) solution into the left side of the CCe in the cerebellum of the adult *Tg(aldoca:TVA-mCherry); Tg(aldoca:G)* fish (*n* = 53, Figure 5). After rearing the fish at 34–35.5°C for 5 days, we fixed the brain and stained sagittal sections with anti-GFP and anti-mCherry antibodies, and a nuclear marker Hoechst. High GFP expression was detected in the PCL and the ML (*n* = 18, Figure 5A). High-magnification images showed that GFP was present in the TVA-mCherry-expressing PCs (Figure 5B), indicating a primary infection of the PCs with EnvA-RVΔG-GFP. In addition to the PCs, GFP was detected in neurons in the GL that were negative for TVA-mCherry (*n* = 6, Figure 5C), indicating a secondary infection with RVΔG-GFP. We found that these neurons were morphologically GCs, since they had a small soma and three or four dendrites. We also detected GFP but not TVA-mCherry in neurons in the contralateral side of the IO (*n* = 1, Figures 5D,E). Considering that CFs project contralaterally from the IO neurons (Takeuchi et al., 2015), these GFP^+^ neurons were probably IO neurons that send CFs to PCs in the cerebellum. To confirm the specificity of these observations, we performed a set of control experiments. When we injected the RV solution into wild-type fish, GFP was not detected (*n* = 4, data not shown). When we injected it into the cerebellum of *Tg(aldoca:TVA-mCherry)* fish, GFP was detected only in PCs (*n* = 5, data not shown), indicating that the TVA-mCherry conferred RV infectivity to the neurons, but G was required for the retrograde transport and the trans-synaptic transmission. Thus, we observed secondary RV infections in the GCs and the IO neurons. The GCs and the IO neurons are major afferent neurons for PCs in all vertebrates. Our data indicated that the RV-mediated trans-synaptic tracing method was applicable in zebrafish.

### Tracing of Precerebellar Neurons for MFs in Zebrafish

We next focused on the MFs by injecting the RV solution into the left side of the cerebellum of the adult *Tg(cbln12:TVA-mCherry); Tg(cbln12:G)* fish (*n* = 58, Figure 6). After rearing the fish at 34–35.5°C for 10 days, we fixed the brains and stained the cross sections with anti-GFP and anti-mCherry antibodies and Hoechst. GFP was detected in the soma and neurites of GCs in the GL in the CCe and in the EG that were also positive for TVA-mCherry (*n* = 13, Figure 6A), confirming the primary infection of the GCs by RV. Since the *cbln12* promoter also drives expression outside the cerebellum, we chose fish that specifically displayed a primary infection of GCs (i.e., having GFP^+^ TVA-mCherry^+^ GCs in the cerebellum) and carefully examined the GFP^+^ (TVA-mCherry^−^, Hoechst^+^) somata outside the cerebellum (*n* = 10). GFP signals were detected in neurons in the pretectal nuclei such as the CPN (*n* = 2, Figure 6B) and the PCN (*n* = 2, Figures 6C,D), and in the medulla oblongata including the (NLV, *n* = 4, Figures 6E–G), the (CG, *n* = 5, Figure 6H), the medial octavolateralis nucleus (MON, *n* = 5, Figure 6I), and the descending octaval nucleus (DON, *n* = 4, Figure 6J). Most of these neurons had a small soma, except for the neurons in the CG, which had a relatively large soma (Figure 6H). TVA-mCherry was not detected in these neurons (Supplementary Figure S2). Although we injected the RV solution into the left side of the cerebellum, there was no difference the number of GFP-positive neurons between the ipsilateral and the contralateral sides. These GFP^+^ neurons were not observed when the RV solution was injected into the *Tg(cbln12:TVA-mCherry)* or the *Tg(aldoca:TVA-mCherry); Tg(aldoca:G)* fish (data not shown), indicating that these neurons receive the virus specifically from GCs. Our data indicated that the brain regions (nuclei) containing GFP^+^ cells were bona fide precerebellar nuclei that sent MFs directly to GCs in the cerebellum.

## Discussion

In this study: (1) we established a transgenic system in which the *cbln12* promoter drives transgene expression in GCs in the cerebellum; (2) we developed an RV tracing method that labeled only the presynaptic neurons in zebrafish; and (3) using our RV tracing method, we identified the bona fide precerebellar nuclei giving rise to MFs in zebrafish.

### *cbln12* Promoter

Many PC-specific genes have been identified in zebrafish (Takeuchi et al., 2017), and the promoter and enhancer elements of some of PC-specific genes, such as *aldoca* (Tanabe et al., 2010) and carbonic anhydrase 8 (*ca8*, Matsui et al., 2014a) have been shown to drive transgene expression in PCs in zebrafish. However, no truly GC-specific genes have been reported, although many genes enriched in GCs have been identified (Takeuchi et al., 2017). Among them, *cbln12* is a zebrafish ortholog of mouse *Cbln1*, which encodes a secreted molecule involved in synapse formation between the axons of GCs and the dendrites of PCs (Uemura et al., 2010; Takeuchi et al., 2017). *Cbln1* is expressed mainly in GCs in the mouse cerebellum (Slemmon et al., 1984; Kusnoor et al., 2010). We previously reported that *cbln12* is expressed in GCs in the TL and the cerebellum (Takeuchi et al., 2017). Consistent with this observation, *Tg(cbln12:Venus)* fish expressed Venus in GCs in the TL and the cerebellum (Figure 2), suggesting that the 2-kbp *cbln12* genomic element was sufficient to drive gene expression in all of the *cbln12*-expressing brain regions. Previous reports showed that there is a cerebellum-like structure in the zebrafish tecum that involves the GCs in the TL (Bell, 2002; Bell et al., 2008; Sawtell and Bell, 2008; Hibi and Shimizu, 2012). Since many of the same GC-enriched genes are expressed in the GCs in the TL and the cerebellum, these cells may share regulatory mechanisms for the expression of these GC genes. Future studies of the *cbln12* promoter may reveal the common mechanisms for gene expression in the GCs.

*cbln12* was shown to be expressed in the telencephalon (Takeuchi et al., 2017), and the *Tg(cbln12:Venus)* fish expressed Venus in the dorsolateral telencephalon (Figure 2). In mice, *Cbln1* is expressed in the retrosplenial granular cortex, and Cbln1 protein is detected in the hippocampus; *Cbln1* in the forebrain is involved in fear conditioning and spatial memory (Otsuka et al., 2016). Considering that the lateral zone of the dorsal telencephalic area (Dl) is likely to correspond to the hippocampus in mice (Ganz et al., 2014), the cbln12:Venus^+^ region may function similarly to *Cbln1*-expressing neurons in the mouse telencephalon. The *cbln12* promoter can be used for studies not only on the cerebellum but also on the telencephalic neural circuits involved in fear conditioning and spatial memory.

The *cbln12* promoter is not a truly GC-specific promoter, although the highest expression driven by this promoter is in the GCs. In this study, the regional injection of the RV solution could have restricted the infection to the GCs in the cerebellum. Similarly, the neuronal activity of GCs can be manipulated by expressing optogenetic tools using *cbln12* promoter-mediated transgenesis and specifically stimulating the cerebellum region with light. Alternatively, combinations of multiple GC-related promoter/enhancers including the *cbln12* promoter and a recombination system (e.g., the Cre-loxP system) could be used to drive transgene expression exclusively in GCs.

### RV Tracing for PC Afferents in Zebrafish

In this study, we demonstrated that retrograde tracing with a pseudotyped RV was applicable in zebrafish. An unpseudotyped recombinant RV was shown to infect neurons in the olfactory bulb and the ventral telencephalon in zebrafish and to induce the expression of a transgene from the RV genome (Zhu et al., 2009). However, tracing with a pseudotyped RV has not been reported in zebrafish. We first showed that after the RV primarily infected TVA-mCherry-expressing PCs, the RV was transferred to the GCs and the contralateral IO neurons (Figure 5), indicating the retrograde, trans-synaptic labeling of the PC afferent neurons. We did not observe any secondary infection to efferent neurons for the PCs, such as the ECs or the neurons in the DON (Bae et al., 2009; Takeuchi et al., 2015). Our data indicated that the RV tracing method detected presynaptic but not postsynaptic neurons in zebrafish, as it does in mouse. In addition to GCs and IO neurons, PCs receive inputs from stellate cells and possibly adjacent PCs (Butler and Hodos, 1996; Altman and Bayer, 1997; Watt et al., 2009); however, we could not identify these neurons by the RV tracing. The inability of detecting stellate cells could be due to the relatively low efficiency of the RV tracing (discussed below) or specific nature of synapses between stellate cells and PCs. In our RV system, we could not distinguish secondary from primary infections of PCs, since TVA-mCherry was expressed in all of the PCs (Figure 3). RV tracing with Tg lines that mosaically express TVA-mCherry and G may be useful for labeling afferent inputs from neighboring PCs.

### Precerebellar Neurons for MFs in Zebrafish

**Figure 7:**
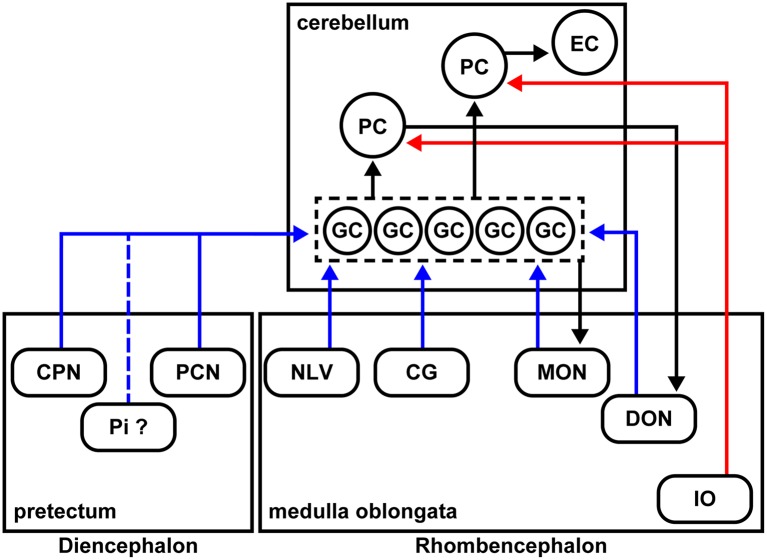
Schematic summary of the cerebellar afferent connectivity in zebrafish. The MFs and climbing fibers (CFs) are indicated in blue and red, respectively. The neural circuits marked by black arrows were reported previously.

We identified many precerebellar nuclei for the MFs (Figure 6). These sites were separately located in the diencephalon and the rhombencephalon (hindbrain; Figure 7). Among them, the CPN and PCN are located in the pretectum region and receive visual information inputs from the retina (Becker et al., 2000; Yáñez et al., 2018). The NLV receive inputs from various sources including the telencephalon and hypothalamus (Yang et al., 2004). Since the pontine nuclei, major precerebellar nuclei for the MFs in mammals, receive projections from the telencephalon, the NLV in teleosts, at least in part, are likely to be functionally equivalent to the pontine nuclei in mammals (Hibi et al., 2017). The CG has been suggested to be involved in several physiological processes, such as the control of sexual behaviors (Ampatzis and Dermon, 2010). The MON and DON are rhombencephalic nuclei that receive lateral line and vestibular sensory information, respectively (Bell, 1981; Edds-Walton, 1998; Mikami et al., 2004; Bell et al., 2008). Our findings regarding the MFs were consistent with the information obtained from non-viral tracing studies in other teleost species (Finger, 1978; Wullimann and Northcutt, 1989; Xue et al., 2004; Folgueira et al., 2006; Huesa et al., 2006). These data indicated that multiple sensory and command signals (possibly from the pretectal nuclei and the NLV) are conveyed by MFs to the GCs in the cerebellum through distinct routes and that they are integrated in GCs or at different levels downstream of the GCs (e.g., the PCs or ECs) with the CF information. Although previous tracing studies showed that the origin of the MFs includes neurons in the Pi in the pretectum and in the LC in the medulla oblongata in zebrafish (Yáñez et al., 2018), neurons in the nucleus tegmentocerebellaris (Uchiyama et al., 1988; Wullimann and Northcutt, 1988, 1989), the nucleus of the commissure of Wallenberg, and the periventricular pretectal nucleus in goldfish (Wullimann and Northcutt, 1989; Xue et al., 2004), we did not detect RV infection (GFP expression) in these regions. We currently do not know why we did not detect these neurons as precerebellar neurons. It might have been due to the relatively low efficiency of the RV tracing (discussed below), or there may be some specificity of the RV infection. Nevertheless, our RV tracing clearly identified bona fide precerebellar nuclei for the MFs in zebrafish.

In mammals, the MFs also convey multiple types of information from different brain regions: telencephalic commands through the pontine nuclei, vestibular information from the vestibular nuclei, and proprioceptive sensory information from the external cuneate nuclei, lateral reticular nuclei, and spinal cord. Therefore, the MFs are at least partly conserved between mammals and zebrafish. In this study, we did not examine the caudal rhombencephalon or the spinal cord. Future studies of the precerebellar neurons in the caudal brain regions (e.g., the spinal cord) and comparative studies with other vertebrates will increase our understanding of the evolution of cerebellar neural circuits. The precerebellar nuclei that were identified in the presynaptic tracing for GCs were not observed in the presynaptic tracing for PCs, indicating that GCs and PCs receive different information as they do in mammals.

Previous studies showed that some MFs project to ipsilateral but not contralateral GCs (Xue et al., 2004; Folgueira et al., 2006). We detected precerebellar neurons for all of the MFs on both the ipsilateral and contralateral sides (Figure 6). The apparent discrepancy can be explained by the nature of the RV infection. The virus can infect GCs through their axons, and the GCs extend their axons bi-laterally to both the left and right sides irrespective of their somal position (Takeuchi et al., 2015). When the virus solution was injected on one side, we observed the primary infection of GCs on both sides (Figure 6A), indicating that the RV infected the GC axons and was transported to the GC somata on both sides. Subsequently, the RV infected precerebellar neurons on both sides. To circumvent this problem, tagging the TVA-mCherry with the Myosin Va-binding domain of Melanophilin may restrict the localization of the TVA-mCherry to the dendrites (Lewis et al., 2009), to prevent axonal infections.

In zebrafish, the crest cells in the MON receive projections from the GCs in the EG and LCa, and the DON neurons receive projections from the PCs located in the caudolateral domains of the cerebellum (Bae et al., 2009; Takeuchi et al., 2015; Figure 7). As in mammals, the zebrafish cerebellum (the ECs) sends outputs to the tegmental red nucleus (Wullimann and Northcutt, 1988; Matsui et al., 2014a,b; Takeuchi et al., 2015); the neurons in the red nucleus project to the IO neurons (Xue et al., 2008; Nakayama et al., 2019). Therefore, the cerebellar neural circuits have multiple feedback mechanisms that are required to control complex behaviors.

### Future Application of the RV Tracing Method

Tools such as HRP, DiI, WGA, and various types of virus have been used for connectome analyses, but RV tracing is the only method that can specifically label presynaptic neurons (Wickersham et al., 2007a,b; Osakada and Callaway, 2013). However, there has been a technical problem with RV tracing in zebrafish. The RVs infect neurons and replicate efficiently at 37°C in mammals, but we normally rear zebrafish around 28.5°C. To alleviate this problem, we reared the RV-injected adult fish at 34–35.5°C for 5–10 days, because they cannot survive at 37°C for 10 days. Even when the fish were reared at the higher temperature, the number of presynaptic neurons marked by GFP expression was small, compared to the numbers of precerebellar neurons labeled in mice. This difference was probably due to both the much smaller number of afferent fibers in zebrafish and the lower efficiency of RV propagation in zebrafish. Quantitative analyses in mice and zebrafish will be required to clarify this issue. The use of different RV strains (Reardon et al., 2016), different types of neurotropic viruses, or the combination of other viruses with the G of RV (Beier et al., 2011), may increase the efficiency of the virus-mediated trans-synaptic tracing in zebrafish.

In this study, we succeeded in identifying cerebellar afferents in serial sections of the fish brain. We did not visualize the entire path of the CFs and MFs or analyze the topographic relationship between the precerebellar nuclei (or neurons) and the cerebellar neurons using whole-mount preparations. Whole-brain connectome analyses combining the RV tracing method and tissue-transparency methods such as CLARITY, CUBIC, and SeeDB (Ke et al., 2013; Susaki et al., 2014; Tomer et al., 2014; Yang et al., 2014) may lead to better determinations of the precise axonal routes of the MFs and a topographic map for the cerebellar neural circuits in zebrafish.

It used to be thought that the GCs receive sparse and temporal information (Marr, 1969; Albus, 1971). However, it was recently reported that a single GC can convey multimodal information (Knogler et al., 2017). We previously reported that in classical fear conditioning, some GCs encode temporal information and are involved in controlling the timing of conditioned responses (Matsuda et al., 2017). It is still not clear whether GCs receive and integrate multimodal signals, or if so, what signals each GC receives. To address this issue, we need to monitor the neuronal activity of the GCs and their presynaptic neurons. A pseudotyped RV with a Ca^2+^ indicator (e.g., GCaMP) in place of the GFP (Osakada et al., 2011; Reardon et al., 2016) could be applied for simultaneous monitoring of the GCs and their afferent neurons.

In any case, the RV tracing method described here provides a new approach for performing neural circuit research in zebrafish.

## Data Availability

All datasets generated for this study are included in the manuscript and/or the Supplementary Files.

## Ethics Statement

The animal work in this study was approved by the Nagoya University Animal Experiment Committee (approval number: 2016022203, 2017030202, 2018031302) and was conducted in accordance with the “Regulations on Animal Experiments in Nagoya University” and “Guidelines for Proper Conduct of Animal Experiments (Science Council of Japan).” The work with transgenic zebrafish and the RV was approved by the Nagoya University Recombinant DNA Experiment Safety Committee and was conducted under the confirmation from the Ministry of Education, Culture, Sports, Science and Technology (MEXT) in Japan.

## Author Contributions

RD performed experiments. MY and FO prepared the RV solution. RD, NY, TS, and MH analyzed the data. MH designed the study, obtained funding for and supervised this study. RD and MH drafted the manuscript.

## Conflict of Interest Statement

The authors declare that the research was conducted in the absence of any commercial or financial relationships that could be construed as a potential conflict of interest.

## Supplementary Material

FIGURE S1 Expression of TVA-mCherry in *Tg(cbln12:TVA-mCherry)* fish. Immunostaining of sagittal sections of the adult *Tg(cbln12:TVA-mCherry)* fish cerebellum with anti-mCherry (anti-DsRed) antibody (magenta). Note that expression of TVA-mCherry was observed in the telencephalon, the mesencephalic tectum and the cerebellum. Expression of TVA-mCherry is similar to that of Venus in the *Tg(cbln12:Venus)* fish (Figure 2F). Scale bar: 200 μm.

FIGURE S2 Expression of GFP but not TVA-mCherry in precerebellar neurons in the RV tracing of MFs. The RV solution was injected into the left side of the cerebellum of adult *Tg(cbln12:TVA-mCherry); Tg(cbln12:G)* fish. The injected fish were then reared at 34–35.5°C for 10 days. The brains were harvested, fixed, and subjected to immunostaining. Staining of brain cross sections with anti-mCherry (magenta) and anti-GFP (green) antibodies, and Hoechst (cyan). **(B’–F’)** Hoechst, GFP, TVA-mCherry, and GFP/TVA-mCherry images of **(B’–F’)** in Figure 6. Scale bar: **(B’)** 50 μm. The magnification in all panels was the same as in **(B’)**.

## Abbreviations

Cb: cerebellum
CC: crista cerebellaris
CCe: corpus cerebelli
CF: climbing fiber
CG: central gray
CPN: central pretectal nucleus
DON: descending octaval nucleus
EC: eurydendroid cell
EG: eminentia granularis
G: envelope glycoprotein
GC: granule cell
GL: granular layer
GoC: golgi cell
IO: inferior olivary nucleus
HRP: horseradish peroxidase
IN: intermediate pretectal nucleus
LC: locus coeruleus
LCa: lobus caudalis cerebelli
Mes: mesencephalon
MF: mossy fiber
MON: medial octavolateralis nucleus
ML: molecular layer
NLV: nucleus lateralis valvulae
PCL: Purkinje cell layer
PCN: paracommissural nucleus
PC: Purkinje cell
PF: parallel fiber
PGZ: periventricular gray zone of the optic tectum
Pi: intercalated pretectal nucleus
RhV: rhombencephalic ventricle
SM: stratum marginale
Tel: telencephalon
TeO: optic tectum
TL: torus longitudinalis
TeV: tectal ventricle
Tg: transgenic
TS: torus semicircularis
Va: valvula cerebelli
Val: lateral lobe of valvula cerebelli
Vam: medial lobe of valvula cerebelli
VAO: ventral accessory optic nucleus.
