# Supplementary figures and images for: Tracing of Afferent Connections in the Zebrafish Cerebellum Using Recombinant Rabies Virus
Source: Front Neural Circuits. 2019 Apr 24;13:30. doi: 10.3389/fncir.2019.00030 (PMC6491863; doi:10.3389/fncir.2019.00030)

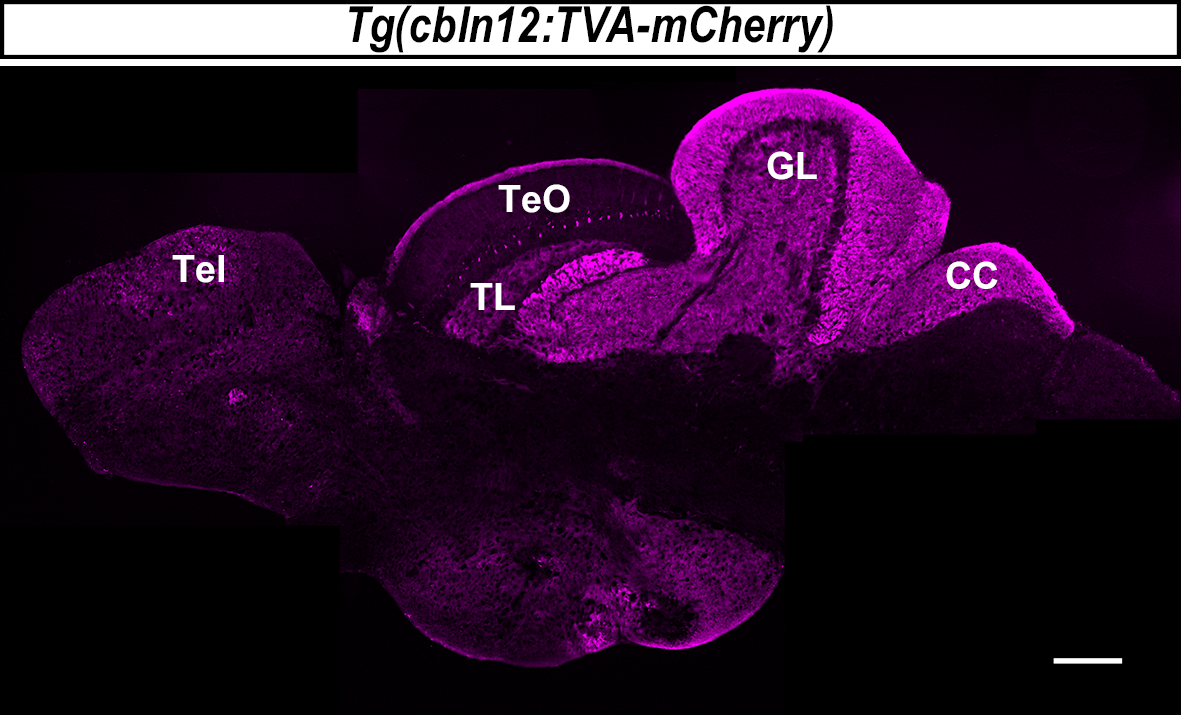

Supplement: FIGURE S1 — Expression of TVA-mCherry in Tg(cbln12:TVA-mCherry) fish. Immunostaining of sagittal sections of the adult Tg(cbln12:TVA-mCherry) fish cerebellum with anti-mCherry (anti-DsRed) antibody (magenta). Note that expression of TVA-mCherry was observed in the telencephalon, the mesencephalic tectum and the cerebellum. Expression of TVA-mCherry is similar to that of Venus in the Tg(cbln12:Venus) fish (Figure 2F). Scale bar: 200 μm. [file Image_1.TIF]

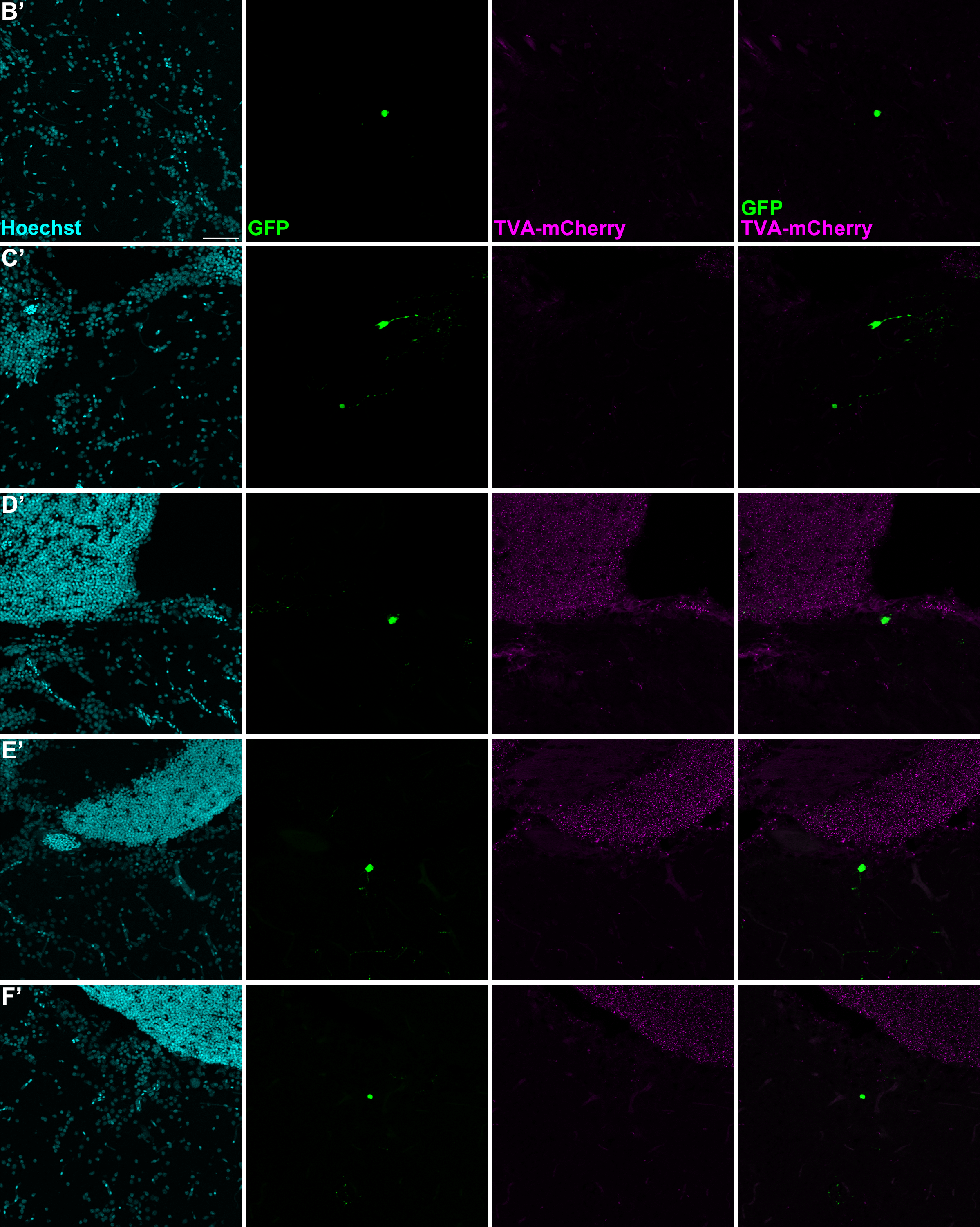

Supplement: FIGURE S2 — Expression of GFP but not TVA-mCherry in precerebellar neurons in the RV tracing of MFs. The RV solution was injected into the left side of the cerebellum of adult Tg(cbln12:TVA-mCherry); Tg(cbln12:G) fish. The injected fish were then reared at 34–35.5°C for 10 days. The brains were harvested, fixed, and subjected to immunostaining. Staining of brain cross sections with anti-mCherry (magenta) and anti-GFP (green) antibodies, and Hoechst (cyan). (B’–F’) Hoechst, GFP, TVA-mCherry, and GFP/TVA-mCherry images of (B’–F’) in Figure 6. Scale bar: (B’) 50 μm. The magnification in all panels was the same as in (B’). [file Image_2.TIF]
